# Application of community phylogenetic approaches to understand gene expression: differential exploration of venom gene space in predatory marine gastropods

**DOI:** 10.1186/1471-2148-14-123

**Published:** 2014-06-05

**Authors:** Dan Chang, Thomas F Duda

**Affiliations:** 1 Department of Ecology and Evolutionary Biology and Museum of Zoology, University of Michigan, Ann Arbor, MI, USA; 2 Department of Statistics, University of Michigan, Ann Arbor, MI, USA; 3 Smithsonian Tropical Research Institute, Ancόn, Balboa, Republic of Panama; 4 Current address: 1156 High Street- mail stop EEBiology, Santa Cruz, CA 95064, USA

## Abstract

**Background:**

Predatory marine gastropods of the genus *Conus* exhibit substantial variation in venom composition both within and among species. Apart from mechanisms associated with extensive turnover of gene families and rapid evolution of genes that encode venom components (‘conotoxins’), the evolution of distinct conotoxin expression patterns is an additional source of variation that may drive interspecific differences in the utilization of species’ ‘venom gene space’. To determine the evolution of expression patterns of venom genes of *Conus* species, we evaluated the expression of A-superfamily conotoxin genes of a set of closely related *Conus* species by comparing recovered transcripts of A-superfamily genes that were previously identified from the genomes of these species. We modified community phylogenetics approaches to incorporate phylogenetic history and disparity of genes and their expression profiles to determine patterns of venom gene space utilization.

**Results:**

Less than half of the A-superfamily gene repertoire of these species is expressed, and only a few orthologous genes are coexpressed among species. Species exhibit substantially distinct expression strategies, with some expressing sets of closely related loci (‘under-dispersed’ expression of available genes) while others express sets of more disparate genes (‘over-dispersed’ expression). In addition, expressed genes show higher *d*_N_/*d*_S_ values than either unexpressed or ancestral genes; this implies that expression exposes genes to selection and facilitates rapid evolution of these genes. Few recent lineage-specific gene duplicates are expressed simultaneously, suggesting that expression divergence among redundant gene copies may be established shortly after gene duplication.

**Conclusions:**

Our study demonstrates that venom gene space is explored differentially by *Conus* species, a process that effectively permits the independent and rapid evolution of venoms in these species.

## Background

Gene regulation shapes inter- and intraspecific phenotypic variation and affects organismal responses to changes in environmental conditions [1]. Vast phenotypic and behavioral differences among closely related species can be attributed to differences in gene regulation [2-6]. Gene expression variability also facilitates individuality of organisms and phenotypic differences among individuals with identical genotypes [7]. Differences in gene expression patterns can be viewed as differential exploitation of ‘gene space’ (i.e., all protein-coding genes) [8]. Diversity and quantities of messenger RNA (mRNA) transcripts of genes in the gene space reflect the functional and adaptive roles of the gene products and represent organismal responses to environmental perturbations in real-time [1].

Gene families are important components of genomes; expression divergence of members of gene families contributes to interspecific differential expression [9-12]. Venoms of many organisms are composed of various potent toxins that are encoded by many gene families, and Lluisma et al. [13] suggested that these organisms differentially explore these ‘venom gene spaces’ (i.e., the combinations of toxin-coding genes in the genome of each species). In particular, some species may fully explore this space (e.g., express a disparate set of available loci), while others may focus within a specific region of the space. This hypothesis stems from observations that numbers and combinations of expressed venom genes differ among species of predatory marine gastropods (*Conus* species) and that genes expressed in certain species do not appear to be random subsets of available genes [13].

Predatory marine snails of the genus *Conus* utilize venoms that include a variety of peptide neurotoxins (conotoxins or conopeptides) that are encoded by various large gene superfamilies and target diverse sets of ion channels and neuronal receptors in prey [14]. Venom composition varies dramatically among and within *Conus* species [15-18], which, in part, derives from the dynamics of conotoxin gene family evolution through extensive gene turnover and rapid evolution [19,20]. Previous studies revealed the importance of differential expression in interspecific divergence of venoms based on analyses of conotoxin gene transcripts [21-25]. In addition, closely related species tend not to express orthologous counterparts, a phenomenon that contributes to interspecific differences in venom composition [23].

Without knowledge of the genomic composition of venom gene space, it is difficult to differentiate transcriptional variation of single genes from lineage-specific gene duplication/loss, especially under scenarios of extensive turnover of conotoxin gene families [19]. Previous descriptions of genomic profiles of A-superfamily loci of four closely-related species, *C. lividus*, *C. sanguinolentus*, *C. diadema* and *C. quercinus*, [19] provide a great opportunity to examine expression patterns of members of this gene family in these species. A-superfamily genes encode α-conotoxins that are selective blockers of nicotinic acetylcholine receptors and characterized by a signature cysteine backbone of “**CC**(X)_m_**C**(X)_n_**C**” [26]. A-superfamily genes possess a highly conserved prepro region (the N-terminus of the translated prepropeptide that is cleaved from the mature toxin following translation) and a fairly conserved 3’ untranslated region that together flank the toxin-coding region [26,27]; the conserved nature of these regions makes it possible to retrieve most if not all members of this superfamily from venom duct transcripts through amplification of cDNA with ‘universal’ primer pairs designed within these regions.

**Figure 1:**
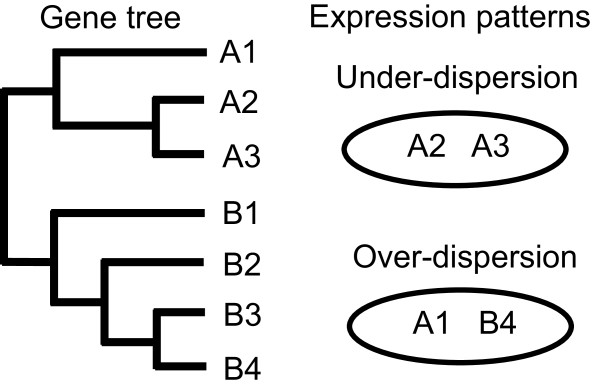
**Schematic of modes of expression patterns of genes in a single species.** On the left is a hypothetical gene tree for seven members of a gene family that occur in the genome of a species. Genes in each clade (A and B) are labeled with the clade name and a number (e.g. genes A1, A2 and A3 in clade A). The circles on the right indicate possible scenarios for gene expression in the species (if only two genes are expressed). Expression of the A2 and A3 genes simultaneously represents the scenario of under-dispersed expression in which the sequence disparity of these two genes is smaller than random; expression of the A1 and B4 genes represents over-dispersed expression because of the disparity of these two genes.

Here we evaluated patterns of conotoxin gene expression of four closely-related *Conus* species and determined how these species differentially exploit their venom gene space. Differential exploration of the venom gene space was inferred previously by Lluisma et al. [13] from observations of the presence/absence of specific sets of gene transcripts in the venom duct expression profiles of *Conus* species, without knowledge of the presence/absence of those genes in the genomes of these species. Here we propose a new approach that takes into account the evolutionary history of gene families to quantitatively evaluate expression patterns of members of a gene family that encode part of the venom gene space. This approach is applicable to other non-venom-related multi-gene families to test modes of evolution of gene expression profiles among species. Our approach was developed from community phylogenetic methodologies [28-30] and classifies patterns of gene family expression into three states: “over-dispersion” (expression of a non-random set of phylogenetically distantly-related gene members), “under-dispersion” (expression of a non-random set of closely related genes) and “neutral” (expression of a random set of genes; Figure 1). Details about this approach are described in the Methods section.

We also examined the selectivity of expressed genes and the role of expression in the evolution of gene families, as well as the relationship between gene duplication and expression divergence. Gene duplication plays an important role in the development of expression patterns of members of a gene family. For example, gene duplication promotes expression divergence of gene copies [31] that affects the retention and functionalization of redundant gene duplicates [32,33]. Divergence in expression of paralogous genes is positively correlated with ages of genes [34], and is likely affected by changes of cis- and trans-regulatory elements [35-37]. Closely-related paralogs show equivalent or less resemblance in patterns of expression than distantly-related genes [38], and divergence in gene expression can be rapidly established among young duplicates [34]. Is this pattern (i.e., the rapid establishment of expression divergence of paralogous genes) also applicable to A-superfamily conotoxin genes? To address these themes, we obtained expression profiles of A-superfamily from venom duct transcripts of four *Conus* species, compared the results with genomic compositions of this gene family in each species, statistically evaluated phylogenetic structures of gene expression among species, and assessed patterns of expression and neutrality of gene duplicates.

## Methods

### Specimens

We obtained specimens of *Conus lividus* (from Hawaii), *Conus diadema* (from Panama) and *Conus sanguinolentus* (from American Samoa) from the Mollusk Division collections at the University of Michigan Museum of Zoology. Specimens of *Conus quercinus* (from Hawaii) were provided by Jon-Paul Bingham (University of Hawaii). Permission to work with these specimens was granted by the curator (Thomas F. Duda, Jr.) at the University of Michigan Museum of Zoology. Venom ducts of these specimens were preserved in RNAlater (Ambion, Inc.) and stored at -20°C and then -80°C.

### Recovery of A-superfamily genes from venom duct transcripts

We extracted mRNA from venom ducts of two individuals each of *C. lividus*, *C. diadema* and *C. quercinus* and one individual of *C. sanguinolentus*, and prepared cDNA following the protocol described in Duda and Palumbi [20]. In brief, we digested venom duct tissue and released mRNA in a ‘binding-washing’ buffer (0.14 M NaCl, 1.5 mM MgCl_2_ and 10 mM Tris HCl, pH 8.6) and 0.5% NP40 detergent, isolated mRNA through use of biotinylated oligo-dT that were bound to streptavidin-coated magnetic beads, and synthesized cDNA from the recovered mRNA.

In an attempt to recover all A-superfamily gene sequences from the venom duct transcripts, we used a set of ‘universal’ primers for A-superfamily gene sequences (forward primer: 5’ATGGGCATGCGGATGATGTTCAC 3’; reverse primer: 5’ GTCGTGGTTCAGAGGGTCCTGG 3’) that anneal to the highly conserved prepro and 3’ untranslated regions respectively. We performed amplifications with venom duct cDNA of each individual, cloned amplification products, and screened and sequenced expected inserts following the approach described by Chang and Duda [19]. We repeated this whole procedure for each individual to help identify non-artefactual sequences (as described in the next section). We generated sequence diversity curves [23] for each individual for each round of amplification to determine if we had adequately surveyed the diversity of expressed A-superfamily transcripts.

### Determination of transcribed loci

We examined sequence chromatograms in Sequencher v4.8 (Gene Codes Corporation) and manually aligned sequences in SE-AL v2.0 [39] based on similarities of nucleotide and translated amino acid sequences (especially the cysteine backbone of α-conotoxins as described by Chang and Duda [19]). We determined non-artefactual sequences by comparing sequences recovered from the two rounds of amplification with sequences previously recovered from the genomes of each species ( [19]; GenBank Accession Numbers JF723384-JF723491); we designated sequences recovered from both rounds of amplification or from both venom duct cDNA and genomic DNA of each species as expressed non-artefactual sequences. We constructed a neighbor-joining tree of all sequences (including artefactual sequences) with the K80 [40] model in PAUP 4.0 [41] to ensure that each major clade contained at least one non-artefactual sequence and be confident that artefactual sequences represent sequences that may contain amplification, cloning, or sequencing-induced errors. We allocated artefactual sequences to respective groups (putative expressed alleles) represented by at least one non-artefactual sequence based on their genetic similarities and clustering patterns in the neighbor-joining tree.

### Phylogenetic relationships of expressed genes and tests of differential expression patterns among species

We performed model selection in jModelTest v0.1.1 [42] with non-artefactual gene sequences recovered from venom duct cDNA of the four *Conus* species. We constructed a Bayesian consensus phylogeny of these sequences with MrBayes v3.1.2 [43] (10,000,000 generations, four Markov chains, two runs and 25% burnin) using the best model HKY [44] + I and one A-superfamily gene sequence from *Conus catus* to root the tree (GenBank accession number FJ868066).

We quantified absolute levels of expression of each allele in each individual with counts of sequenced colonies containing inserts of that expressed allele and its respective artefactual sequences, and quantified levels of expression of each locus by combining counts of all alleles of that locus. We pooled expression data of two individuals of *C. lividus*, *C. diadema* and *C. quercinus* to represent expression profiles of these species. To standardize levels of expression among species we calculated relative expression of each locus of each species by dividing total counts of that locus with total counts of colonies sequenced for that species. We examined the numbers and expression levels of orthologous genes coexpressed between species.

### Evaluation of conotoxin gene expression patterns

We developed an approach of evaluating patterns of gene family expression in each species from community phylogenetic methodologies [28-30] that are used to assess phylogenetic diversities of ecological communities. Webb et al. developed two parameters, Net Related Index (NRI) and Nearest Taxon Index (NTI), to quantify patterns of species distributions among different communities [30]. NRI represents standardized differences of the observed mean phylogenetic distances (MPD) between observations and a null model. NTI is the standardized difference of Mean Nearest Taxon Distances (MNTD) for the sample community and a null model. The null model assumes that a community is composed of a phylogenetically random set of species from a species pool (combination of species within a large geographic region enclosing multiple communities). Two alternative types of community assemblies were proposed based on the phylogenetic relatedness of their species components: an over-dispersed community composed of a non-random set of distantly related species, and an under-dispersed community comprised of a non-random set of closely related species [30] (Figure 1).Here we modified this approach to organize and characterize patterns of expression of genes of single species. This approach takes into account phylogenetic signals of genomic profiles, accepts input of lists of expressed genes, and evaluates the distribution of expressed genes in the genealogy of genomic components of gene families within a single species. We regard the gene repertoire of a conotoxin superfamily in the genome of each species (i.e., the ‘gene pool’) as being equivalent to the species pool, and genes expressed in the venom duct of that species to represent to the community species assembly. The inferred genealogy of all members of this gene family is analogous to the phylogeny of the species pool. Two non-random expression patterns based on genealogical structure of expressed genes are then distinguished: under-dispersed and over-dispersed expression (Figure 1). We used mean genetic distance (MGD), mean nearest gene distance (MNGD), nearest gene index (NGI), analogous to MPD, MNTD and NTI, as well as net related index (NRI), to quantify the phylogenetic similarities of expressed genes. MGD and MNGD values for the null model are estimated through random drawing of a specific number of genes from the gene pool, and are then compared with the observed MGD and MNGD values. NRI and NGI represent standardized differences of MGD and MNGD values between the null model and observation. Observed MGD and MNGD values that are less than those obtained through random draws as well as positive NRI and NGI values suggest under-dispersion of gene expression, while observed MGD and MNGD values that are greater than those obtained through random draws and negative NRI and NGI values suggest over-dispersion. Significance of results is determined through non-parametric methods by estimating the percentages of random drawings that yield MGD and MNGD values that are greater than observed values.

We analyzed patterns of gene expression with community phylogenetic approaches with the software package Phylocom [29]. To build separate genealogies for each species, we pruned the phylogeny of conotoxin genes recovered from genomic DNA of the four species (obtained from [19]) with Maximum-Likelihood and HKY + G model in PAUP 4.0 [41]. For each species we imported the pruned genealogy of A-superfamily genes along with a list of expressed genes into Phylocom and evaluated the phylogenetic structure of these expressed genes with the *Comstruct* command and the null model set to 0 and generations to 10,000. This command samples the same number of expressed genes from the gene pool in the genome of each species randomly for 10,000 times, calculates MPD and MNTD values for each random sample, constructs a ranking of simulated results, and determines the significance of the observation based on its ranking among simulated results. The MPD, MNTD, NRI and NTI values produced by this analysis are the values of MGD, MNGD, NRI and NGI indices used in our approach.

### Estimation of ω of expressed genes

We used a maximum-likelihood approach and branch-site model implemented in the Codeml package of PAML 4.3 [45] to test the neutrality of expressed A-superfamily genes. We used this method to determine if ω values (*d*_N_/*d*_S_) of branches leading to expressed A-superfamily genes are significantly different from ω values of branches associated with genes that are not expressed. We excluded sequences of putative pseudogenes as well as a short sequence (livi_51, a α4/3 type conotoxin) from analyses to enable analyses of complete toxin-coding regions. We set one ω value across the whole tree as the null model and proposed three alternative models. The first model assumes that branches leading to expressed genes exhibit a different ω value from that of branches leading to unexpressed and ancestral gene sequences (ω_2_ for terminal branches of expressed loci, ω_1_ for the rest of the branches). The second model assumes the opposite and is different from the first model in that it groups branches leading to expressed genes with internal branches (ω_2_ for the terminal branches of unexpressed genes, ω_1_ for the rest of the branches). The third model assumes that branches leading to expressed, unexpressed and ancestral genes exhibit different ω values respectively (ω_1_ for ancestral branches, ω_2_ for terminal branches of unexpressed genes, ω_3_ for terminal branches of expressed genes). We also used a full model permitting variable ω values for each branch in the genealogy. *P*-values were estimated with likelihood-ratio tests of the null model with alternative models.

### Expression divergence of gene duplicates

We compared relative expression levels of inparalogous genes (paralogous genes generated from lineage-specific gene duplication [46]) within each species. We investigated the relationships between expression divergence of conotoxin genes and time of divergence and rates of evolution of these genes. Divergence time between paralogous genes is represented by the number of synonymous substitutions per synonymous site (*d*_S_) between pairs of paralogs, while rates of evolution are approximated with ω (*d*_N_/*d*_S_). We estimated pairwise *d*_S_ (based on prepro and toxin-coding regions) and *d*_N_ values (based on toxin-coding regions) of A-superfamily genes that were previously recovered from the genome of each *Conus* species in MEGA v5.05 using the Nei-Gojobori method with Jukes-Cantor correction [47]. For gene pairs with *d*_S_ = 0, we converted these zero-value *d*_S_ estimates to 0.004 to avoid derivation of values of infinity for the ω values (the synonymous substitution rate of conotoxin genes is approximated with the synonymous substitution rate of the β-tubulin gene—0.004 per million years—as described by Chang and Duda [19]).

Previous studies have designated expression divergence of gene duplicates as fold-changes of expression levels based on results from microarray analyses [34,38], but this approach is not applicable to our study because our expression data were based on presence/absence of sequences recovered from cDNA libraries. Thus to compare patterns of expression, we grouped observations into three discrete categories: cases in which (i) both paralogs are not expressed, (ii) one gene is expressed and the other is not, and (iii) both genes are expressed. We compared *d*_S_ and ω values among the three categories and tested if the mean values of ω are significantly different between categories with t-tests and ANOVA in R v2.15.0 [48]. All scripts used in this study are available upon request (from DC).

## Results

### Percentages of A-superfamily genes expressed in each species

We sequenced 487, 167, 135 and 112 colonies from two individuals each of *C. lividus*, *C. diadema*, *C. quercinus* and one individual of *C. sanguinolentus* (Table 1). After identification and elimination of artefactual sequences, we determined 18, 3, 4 and 5 putative alleles for each species (alignments of unique sequences and putative alleles are included as the Additional file 1 and Additional file 2). All artefactual sequences appear to represent sequences with amplification or cloning-induced errors. The non-artefactual alleles were all retrieved from the genome of each species previously [19]. Based on comparison of these alleles with A-superfamily genes identified from genomic DNA of these species [19], the alleles represent 13 loci in *C. lividus*, three in *C. diadema*, three in *C. quercinus* and five in *C. sanguinolentus* (Table 1). Based on the number of A-superfamily genes previously reported from the genomes of each species (32 genes in *C. lividus*, 18 in *C. diadema*, 12 in *C. quercinus* and 18 in *C. sanguinolentus*), 40.6% of A-superfamily genes in *C. lividus*, 16.7% in *C. diadema*, 25.0% in *C. quercinus* and 27.8% in *C. sanguinolentus* are expressed in venom ducts of these species (Table 1).

**Table 1:** Expressed A-superfamily conotoxin recovery information

	***C. lividus***	***C. diadema***	***C. quercinus***	***C. sanguinolentus***
**Colonies sequenced**	487	167	135	112
**A-superfamily sequences**	459	156	107	100
**Unique sequences**	66	26	17	26
**Alleles**	18	3	4	5
**Loci**	13	3	3	5
**Fraction of genes in the genome that are expressed**	40.6%	16.7%	25.0%	27.8%

Numbers of colonies screened and sequenced, putative A-superfamily gene sequences, unique sequences, non-artefactual alleles and loci recovered from venom duct transcripts of *Conus* species, as well as the percentages represented by expressed genes in the genomic profiles of A-superfamily of each species. Alignments of unique sequences and alleles are shown in Additional file 1 and Additional file 2.

### Diversity of expressed genes

**Figure 2:**
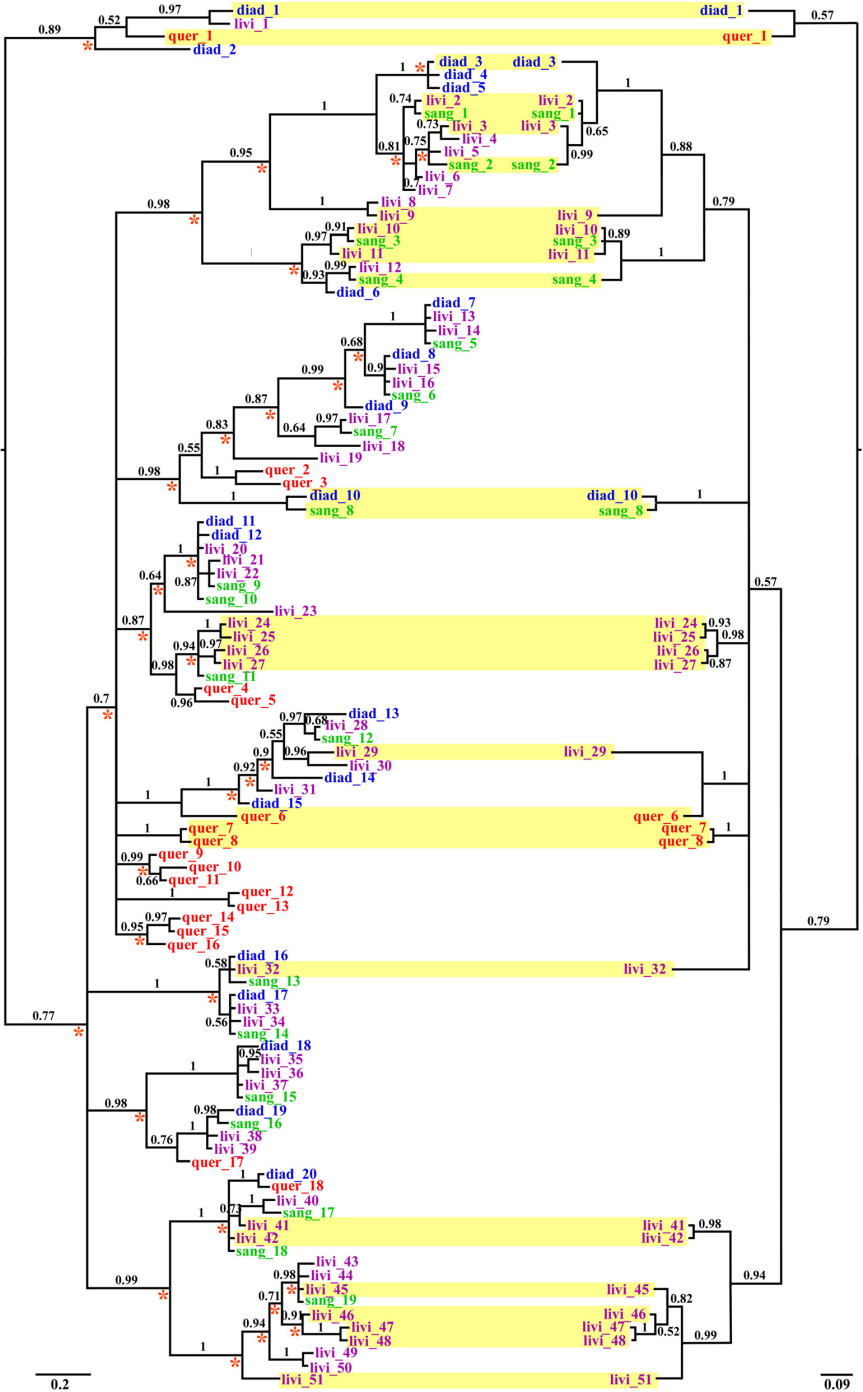
**Phylogenies of A-superfamily conotoxin genes retrieved from genomic DNA and venom duct cDNA of four *Conus *species.** Bayesian consensus phylogeny of putative allele sequences of all genes recovered from the genomic DNA of these species (i.e., ‘the genome phylogeny’) constructed with complete deletion and the HKY + I + G model (left). Bayesian consensus phylogeny of putative allele sequences expressed in venom ducts of these species (i.e., ‘the expression phylogeny’) constructed with complete deletion and the HKY + I model (right). Posterior probabilities are labeled at each node. Sequences that are expressed are shaded in yellow in both trees; putative duplication events are labeled with red asterisks in the genome phylogeny.

Out of the 24 loci expressed by these four *Conus* species, 22 appear to represent functional genes because predicted amino acid sequences represent potentially active α-conotoxins based on the presence of an intact cysteine framework. Previously we discovered three types of pseudogenes of A-superfamily gene sequences recovered from genomes of these four species [19]: pseudogenes of types I and II contain premature stop codons in the toxin coding regions and those of the type III have one non-synonymous substitution in the fourth cysteine codon position of the cysteine backbone. Here we found that three unique alleles of two loci representative of the type III pseudogenes are expressed exclusively in *C. lividus* (Figure 2), and the other pseudogene types are not expressed. A-superfamily genes of these species encode four types of α-conopeptides (α4/4, α4/7, α4/6 and α4/3) [19], among which genes of the α4/7 type dominate both genomic and transcriptomic repertoires (Figure 2). One of the three loci of the α4/6 type and the only locus of the α4/3 type that were exclusively found in *C. lividus* are expressed. An α4/4 type locus that was characterized from the genomes of *C. diadema*, *C. quercinus* and *C. lividus* and that presumably represents an orthologous counterpart in these species was recovered from cDNA of *C. diadema* and *C. quercinus* but not *C. lividus* (Figure 2).

### Limited coexpression of orthologous genes

Similarity in expression patterns of species can be represented by the numbers of orthologous loci that are coexpressed by these species [23]. Only a few orthologous loci are coexpressed by the four *Conus* species examined here, and no orthologous counterparts are expressed simultaneously by more than two species (Table 2). *C. lividus* does not coexpress any gene in common with *C. diadema* or *C. quercinus*, while *C. diadema* only expresses one orthologus counterpart with *C. sanguinolentus* (diad_10 and sang_8) and *C. quercinus* (diad_1 and quer_1) (Figure 2; Table 2). Only two orthologous counterparts were recovered from *C. lividus* and *C. sanguinolentus* (livi_2 and sang_1; livi_10, livi_11 and sang 3; Figure 2; Table 2), even though these two species diverged less than 0.3 million years ago and may actually represent genetically differentiated populations of *C. sanguinolentus*[19,49]. Sequences of these orthologs are identical (i.e., sequence livi_2 is the same as sang_1 and livi_10 is the same as sang_3), which suggests recent divergence of these species. Moreover, the four orthologous genes coexpressed by multiple species exhibit considerable heterogeneity in expression levels (Additional file 3: Figure S1A).

**Table 2:** Numbers of orthologous loci that are coexpressed among species (below diagonal) and their proportions in the venom duct expression profiles of each species (above diagonal)

	***C. lividus***	***C. diadema***	***C. quercinus***	***C. sanguinolentus***
***C. lividus***	-	0/0	0/0	15.4/40.0
***C. diadema***	0	-	33.3/33.3	33.3/20.0
***C. quercinus***	0	1	-	0/0
***C. sanguinolentus***	2	1	0	-

The number before the forward slash in each cell is the percentage (%) of coexpressed loci in the species of the row label of the cell, and the number after the slash is the percentage in the species of the respective column label.

### Differential exploration of venom gene space

Estimation of patterns of expression with the modification of the community phylogenetics approach revealed contrasting results for the four *Conus* species examined. Gene expression patterns of *C. lividus* and *C. sanguinolentus* exhibit MGD and MNGD values that are less than those calculated for the null model as well as positive NRI and NGI values, while the opposite results were detected for expression patterns of *C. diadema* and *C. quercinus* (Table 3). Significance (i.e., *P-*value < 0.5) is only reached for *C. diadema* and *C. sanguinolentus* (Table 3).

**Table 3:** Community phylogenetic indices as evaluations of phylogenetic structure of expressed genes

**Species**	**MGD observed**	**MGD null**	**NRI**	**MNGD observed**	**MNGD null**	**NGI**
*C. lividus*	0.597 ^*P*=0.466^	0.599	0.058	0.193 ^*P*=0.337^	0.213	0.431
*C. sanguinolentus*	0.503 ^*P*=0.056^	0.612	1.678	0.186 ^*P*=0.023^	0.415	2.231
*C. diadema*	0.850 ^*P*=0.016^	0.572	-2.281	0.758 ^*P*=0.021^	0.487	-1.902
*C. quercinus*	0.506 ^*P*=0.098^	0.352	-1.361	0.410 ^*P*=0.120^	0.289	-1.168

Mean geneticdistance (MGD), net relatedness index (NRI), mean nearest phylogenetic gene index (MNGD) and nearest gene index (NGI) were estimated for each species. 10,000 generations of simulations of random sampling of the phylogenetic tree of each species were performed and *P*-values were determined by percentages of random samples smaller or larger than observations. MGD and MNGD values for the null model are averages of 10,000 generations of simulations.

### ω values of contemporaneously expressed genes

Expressed conotoxin genes exhibited a larger ω value than those that are not expressed and inferred ancestral genes. The first alternative model with two ω rates (ω2 for expressed terminal branches and ω1 for the rest of branches in the genealogy) is significantly better than the null model that assumes the same ω value across the whole phylogeny, and ω2 is much greater than ω1 (Table 4). Assigning three free ω variables to the genealogy (ω_1_ for ancestral branches, ω_2_ for terminal branches of unexpressed genes, ω_3_ for terminal branches of expressed genes) showed no significant improvement in likelihood scores, but expressed genes still maintain a greater ω value (Table 4). Moreover, when expressed terminal branches share the same ω as the ancestral branches, the ω value of expressed genes is still greater than that of the contemporaneously non-expressed terminal branches, though this model showed no significant improvement from the null model (Table 4). These results consistently revealed heightened ω values of terminal branches leading to expressed genes, a pattern that still holds when we examined genes of individual species separately (Additional file 3: Table S1).

**Table 4:** **Models used to test if presently expressed genes exhibit higher ω (*d***_**N**_**/*d***_**S**_**) values than the rest of the genes and results of the tests**

**Model**	**ω**	**Ln(*L*)**	***P-value***
Null: One rate	ω = 1.645	-1091.238	-
Alternative: Two rates	ω_1_ = 1.418, ω_2_ = 7.813	-1089.095	0.038
Alternative: Two rates reversed	ω1 = 1.642, ω2 = 1.653	-1091.238	1.000
Alternative: Three rates	ω1 = 1.332, ω2 = 1.651, ω3 = 7.824	-1088.990	0.106

Ln(*L*): log-likelihood of each model. *P*-values were estimated with the Likelihood Ratio Tests of null and alternative models. Definitions of ω variables in each model are described in the methods section.

### Relationships of expression divergence of conotoxin genes duplicates with divergence time and rates of evolution

We tested if expression divergence of conotoxin gene duplicates is affected by the divergence time after gene duplication, and if this expression divergence affects rates of evolution of these genes. We used rates of synonymous substitution (*d*_S_) to approximate to the divergence time of conotoxin paralogs, and ω to approximate rates of evolution of these genes. We categorized expression divergence among species as follows: (i) pairs of genes are not expressed, (ii) only one gene is expressed and (iii) both genes are expressed. Average *d*_S_ and ω values are nearly identical among genes representing the three categories for *C. diadema*, *C. sanguinolentus* and *C. quercinus*; Analysis of Variance (ANOVA) analyses did not reveal any significant differences (Additional file 3: Figure S2). We also combined pairs of genes of categories i and iii (both paralogous gene pairs are either unexpressed or expressed simultaneously) into a group of ‘no expression divergence’, and viewed category ii (only one gene in the gene pairs is expressed) as a group of ‘expression divergence’, to eliminate the possible impact of sample size biases among the three categories on significance of the results (Additional file 3: Figure S2). Student’s t-tests revealed no significant difference in *d*_S_ and ω between these two groups. As an exception, ANOVA analyses and t-tests showed no difference in average ω values among categories or between groups for *C. lividus*. But average *d*_S_ values for categories ii and iii (only one gene or both genes in the gene pairs are expressed) are significantly smaller than category i (neither of gene pairs are expressed) (ANOVA results: estimated difference of mean *d*_S_ between category i and ii is -0.051, *P*-value < 0.0001; estimated difference of mean *d*_S_ between category i and 3 is -0.095, *P*-value < 0.0001; Additional file 3: Figure S2); the two groups of expression defined here (‘no expression divergence’ vs ‘expression divergence’) show no significant differences (*P*-value = 0.0796). Similarly, the average ω value for category ii is significantly greaterthan category i (ANOVA: estimated difference between categories is 2.554, *P*-value = 0.03).

Genes that originate from lineage-specific duplications (defined as inparalogs by Koonin [46]) exhibit discordant patterns of expression: most inparalogs are either not expressed or expressed at different levels. Four genes recovered from *C. lividus* (livi_24 and livi_26; livi_46 and livi_47) and two genes from *C. sanguinolentus* (sang_3 and sang_4) represent three sets of inparalogs that are expressed simultaneously (Figure 2), while no inparalogs were retrieved from *C. diadema* and *C. quercinus*. Moreover, relative expression levels differ vastly between inparalogs that are expressed contemporaneously in *C. lividus* and *C. sanguinolentus* (Additional file 3: Figure S1B).

## Discussion

We investigated patterns of interspecific variation in expression of A-superfamily conotoxin genes in venom ducts and strategies of gene expression of four closely related *Conus* species. Results revealed a remarkable pattern of partial and differential expression of conotoxin genes among and within species and that species exhibit a variety of expression patterns including over-dispersed and under-dispersed expression of gene families. Our study demonstrates that variation in gene expression patterns, combined with the rapid evolution of toxin-coding gene sequences, has contributed to tremendous differences in venom composition among species.

### Partial and differential expression of conotoxin genes among species

Only a subset (less than 50%) of A-superfamily genes that were previously found in the genomes of the four target species were expressed in the individuals we examined. This phenomenon, in part, may be related to the functional fates of these genes. For example, genes that were not expressed may be pseudogenized or in the process of pseudogenization, but this scenario seems unlikely because the majority of unexpressed genes appear to encode functional α-conotoxins [19]. Alternatively, conotoxin genes may perform different roles during ontogeny such that some genes are up-regulated or exclusively expressed in juvenile or subadult developmental stages (Chang and Duda under review); environmental and physiological conditions may affect conotoxin gene expression as well.

Based on the expression patterns detected, A-superfamily conotoxin genes appear to be differentially regulated among species. There is only very little to no overlap in expressed genes among species, even between sister species that diverged very recently (Table 2). Limited coexpression of orthologous conotoxin genes among species has also been inferred for other *Conus* species [20,23]. This pattern suggests that differential expression of conotoxin genes is a prevalent mechanism in generating venom diversity among *Conus* species. Although intraspecific variation in venom composition has been observed in several *Conus* species [16-18], this variation appears to be much less than levels of interspecific divergence in gene expression.

### Expression patterns and applicability of a community phylogenetics approach to study gene expression

Results from the community phylogenetic approach show that *Conus* species employ different strategies in exploiting their venom gene space. Expression patterns of *C. lividus* and *C. quercinus* are not significantly different from random (Table 3). Nonetheless, results for both *C. sanguinolentus* and *C. diadema* are significantly nonrandom; this implies that *C. sanguinolentus* expresses an under-dispersed assortment of genes while *C. diadema* more fully explore their venom gene space (i.e., exhibits over-dispersed expression) (Table 3). The community phylogenetics approach proves to be effective in detecting differences in expression patterns among species and is applicable to evaluation of modes of expression of other multi-gene families.

Under-dispersed gene expression patterns are associated with cases when genes originating from recent duplications are more likely to be expressed than distantly related paralogs, and vice versa. Sister species (e.g., *C. lividus* and *C. sanguinolentus*) tend to express genes that originated from relatively recent duplication events, while genes expressed by *C. diadema* and *C. quercinus* appear to have originated from more ancient duplications. In terms of functional diversities of these genes, if the functional disparity of toxins is associated with their sequence disparity, *C. diadema* (which exhibits a significantly over-dispersed expression pattern) produces toxins that are likely to be more functionally diverse than the other species. Different patterns of gene expression among species may also be affected by the numbers of genes expressed by each species, as the species exhibiting over-dispersed expression, *C. diadema*, coincidently expressed fewer genes than the species exhibiting under-dispersed expression (Table 1). These observations imply that the functional diversities of venoms can be achieved through expression of few genes that encompass more complete sampling of venom gene space or expression of many genes that more fully explore subsections of this space which might permit opportunities for fine-tuning the subfunctions of venom components.

The significantly non-random patterns of gene expression in two species and the difference in expression strategies among *Conus* species imply that conotoxin gene expression is affected by selection. We detected strong selection on the contemporaneously expressed genes that exhibit a significantly larger ω value than non-expressed ones (Table 4 and Additional file 3: Table S1). This implies that expression affects gene evolution by differentially regulating exposure of genes to selection. The lower values of ω for unexpressed genes suggest that these genes may be turned off or down-regulated permanently. Otherwise, selection may be highly variable through time (e.g. during ontogeny) such that genes that are switched off temporarily are subject to different levels/types of selection.

On the other hand, expression strategies used by each species may be shaped by interspecific divergence in selective forces. Interspecific differentiation of expression may be affected by genetic drift and selection [50]. The significantly non-random patterns of gene expression in some species (Table 3) and lack of coexpression of orthologous genes between species (Additional file 3: Figure S1A) imply that variation in conotoxin gene expression is not solely due to drift. Because conotoxins are primarily used for predation, interspecific differences in selection pressures likely stem from differences in the diversity and composition of prey of *Conus* species. Previous studies demonstrate that allelic variation of conotoxin genes is positively correlated with dietary diversity [51], and suggest that gene turnover is associated with dietary breadth of species [19]. *C. lividus* and *C. sanguinolentus* possess broader diets than the other two species [19], a pattern that is possibly related to differences in numbers of expressed conotoxin genes in the venoms of these species. In addition, the significantly over-dispersed gene expression observed for *C. diadema* (Table 3), a pattern that is disparate from those of the other species, may be related to geography (*C. diadema* occurs in the eastern Pacific, while the other species occur in the Indo-West Pacific) and the communities of prey that this species encounters.

### Expression divergence of gene duplicates

Because no significant differences in *d*_S_ or ω were detected among categories of expression in three of the four *Conus* species, expression divergence of conotoxin genes does not appear to be closely associated with divergence time or the rates of evolution of these genes. As an exception, the average *d*_S_ value of *C. lividus* is significantly smaller for genes that are differentially expressed than for unexpressed genes (Additional file 3: Figure S1). This implies that paralogous genes that are differentially expressed are younger than pairs of paralogs that are unexpressed simultaneously. Expression divergence is also positively associated with heightened rates of evolution of these genes in this species: average ω values of differentially expressed genes are significantly greater than those of unexpressed genes.

Previous studies present contradictory results concerning the association between expression divergence and sequence differences in coding regions (as an approximation to divergence time): positive correlations were detected in model organisms such as yeast [34,36] and human [52], but not in *Arabidopsis thaliana*[53]. We found that relationships between expression divergence and divergence time differ among species, and such an association was only detected for *C. lividus*. Gene duplication heightens the probability of expression divergence of paralogous genes [31], but expression divergence and sequence distances are only coupled within a short timeframe after duplication [34,38,52]. Here we found that inparalogs of *C. lividus*, *C. sanguinolentus* and *C. diadema* are either not coexpressed or coexpressed at different levels (Figure 2; Additional file 3: Figure S1B). These results imply that expression divergence is established for inparalogs and recent paralogs and support the notion proposed by Gu et al. [34] that expression divergence can be rapidly fixed in recent gene duplicates. Differential expression contributes to the eventual retention and evolution of gene duplicates because mutations in the unexpressed gene copies, temporarily unexposed to purifying selection, accumulate through time. Beneficial mutations, combined with positive selection facilitate the rapid evolution and neofunctionalization of these genes. Admittedly, our approach did not incorporate information on actual expression levels and the arbitrary division of genes into non-numerical categories of expression (see Methods) may affect the ability to detect differences in these levels.

## Conclusion

We demonstrated partial and differential expression of venom genes among *Conus* species, and supported the idea that species differentially explore their venom gene space through over- and under-dispersed expression of the available repertoire of A-superfamily genes that is present in the genome of each species. Expressed genes are subject to strong positive selection, and expression divergence of gene duplicates appears to be established at an early stage. Extensive gene duplication and selection facilitate variation in gene expression and rapid evolution, combinations of which lead to interspecific divergence in venom composition. Our approach of examining patterns of gene expression proves to be effective in evaluating the differential exploration of the venom gene space, and can be widely utilized for investigation of patterns of gene family expression among species.

### Availability of supporting data

The data sets supporting the results of this article are available in the TreeBASE repository, study ID 15827, http://treebase.org/treebase-web/search/study/summary.html?id=15827[54].

## Abbreviations

mRNA: Messenger RNA; cDNA: Complementary DNA; NRI: Net related index; NTI: Nearest taxon index; MPD: Mean phylogenetic distances; MNTD: Mean nearest taxon distances; MGD: Mean genetic distance; MNGD: Mean nearest gene distance; NGI: Nearest gene index; *d*_*N*_: Non-synonymous substitutions per non-synonymous site; *d*_*S*_: Synonymous substitutions per synonymous site; ω: *d*_N_/*d*_S_.

## Competing interests

The authors declare no competing interests.

## Authors’ contributions

DC and TFD designed the project, DC performed the experiments, DC and TFD analyzed the data and wrote the manuscript. Both authors read and approved the manuscript.

## Authors’ information

DC obtained her PhD in Ecology and Evolutionary Biology from the University of Michigan in 2012, under supervision of the senior author TFD, and also obtained a Master’s degree in Statistics simultaneously. Her dissertation is on the evolution and expression of gene families, using conotoxin genes of predatory marine snails *Conus* as the study system. She is currently a postdoctoral research fellow at the University of California, Santa Cruz, where she is investigating ancient DNA, phylogeography of the extinct megafauna and evolutionary genomics of horse domestication. She is specialized in evolutionary genomics, molecular ecology, phylogeography, bioinformatics and statistical modeling.

TFD is an Associate Professor in the Department of Ecology and Evolutionary Biology and an Associate Curator of Mollusks at the Museum of Zoology at the University of Michigan, Ann Arbor. He obtained his PhD from Harvard University in 1999, and worked as a postdoctoral fellow at the Smithsonian Tropical Research Institute and University of Washington before he began his position at the University of Michigan. He investigates themes in marine invertebrate zoology, evolutionary genetics, molecular ecology, population genetics, phylogenetics and biogeography, with a special focus on marine molluscs. Much of his research program focuses on the evolutionary history of cone snails and conotoxin genes.

## Supplementary Material

Additional file 1 Alignment of unique sequences recovered from each species. Click here for file

Additional file 2 **Alignment of expressed alleles.** Names of alleles are consistent with names of sequences in Figure 2. Click here for file

Additional file 3 Supporting table S1 and figures S1–S2. Click here for file
