# Supplementary material for: Application of community phylogenetic approaches to understand gene expression: differential exploration of venom gene space in predatory marine gastropods
Source: BMC Evol Biol. 2014 Jun 5;14:123. doi: 10.1186/1471-2148-14-123 (PMC4064522; doi:10.1186/1471-2148-14-123)

Table S1. Results of maximum-likelihood estimates of ω values for A-superfamily genes of *Conus* species. Gene sets with and without type III pseudogenes were both used for *C. lividus* and *C. sanguinolentus*; a short sequence (livi_51) was also excluded for analyses of sequences of *C. lividus*. The null model included one ω rate across the whole phylogeny while the alternative model used one ω for expressed genes and another one for non-expressed genes. *P*-values were determined by Likelihood Ratio Tests of log-likelihoods of null and alternative models with one degree of freedom.

**Gene Set**	**Model**	**ω**	**Ln(L)**	***P*-value**
C. diadema	Null: One rate<br>Alternative: Two rates	ω= 0.679<br>ω_1=0.624, ω_2= 999	-572.480<br>-570.874	0.073
C. lividus	Null: One rate<br>Alternative: Two rates	ω= 1.429<br>ω_1= 1.181, ω_2= 999	-730.687<br>-727.245	0.008
C. lividus, no pseudogenes	Null: One rate<br>Alternative: Two rates	ω= 1.285<br>ω_1= 1.069, ω_2= 999	-682.324<br>-678.802	0.008
C. quercinus, no pseudogenes	Null: One rate<br>Alternative: Two rates	ω= 1.379<br>ω_1= 1.790, ω_2= 0.939	-300.619<br>-300.305	0.428
C. sanguinolentus	Null: One rate<br>Alternative: Two rates	ω= 1.082<br>ω_1= 0.981, ω_2= 999	-685.390<br>-683.310	0.041
C. sanguinolentus, no pseudogenes	Null: One rate<br>Alternative: Two rates	ω= 0.970<br>ω_1= 0.862, ω_2= 999	-642.142<br>-639.839	0.032

Figure S1. Relative expression levels of coexpressed orthologs between species and inparalogs within species. Names of loci recovered from *C. lividus* begin with the letter L, *C. diadema* with D, *C. quercinus* with Q and *C. sanguinolentus* with S; numbers after the first letter correspond to numbers in the sequence labels given in Figure 1.

(A) Relative expression levels of orthologous conotoxin loci coexpressed by two *Conus* species. Dark and light grey bars represent orthologous loci expressed by each species.

(B) Relative expression levels of coexpressed inparalogs (those apparently generated by lineage-specific gene duplications). Dark and light grey bars represent the paralogous genes expressed within individuals of each species.

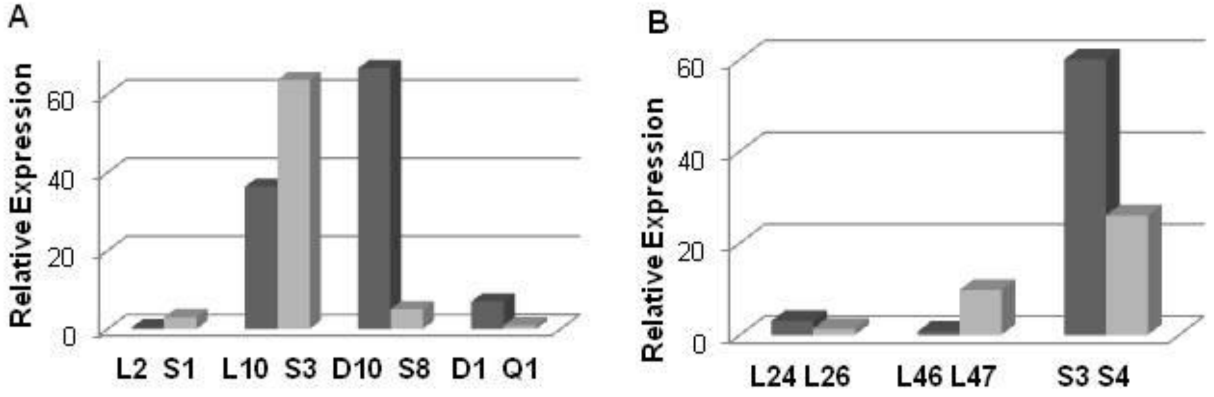

Figure S2. Boxplots of numbers of synonymous substitutions per synonymous site (*d*s, y-axis) against categories of expression (1: neither of the gene pair is expressed; 2: one gene is expressed; 3: both genes are expressed) of four *Conus* species. Sample sizes are labeled next to each category. (A) *C. lividus*, (B) *C. sanguinolentus*, (C) *C. diadema*, and (D) *C. quercinus*.

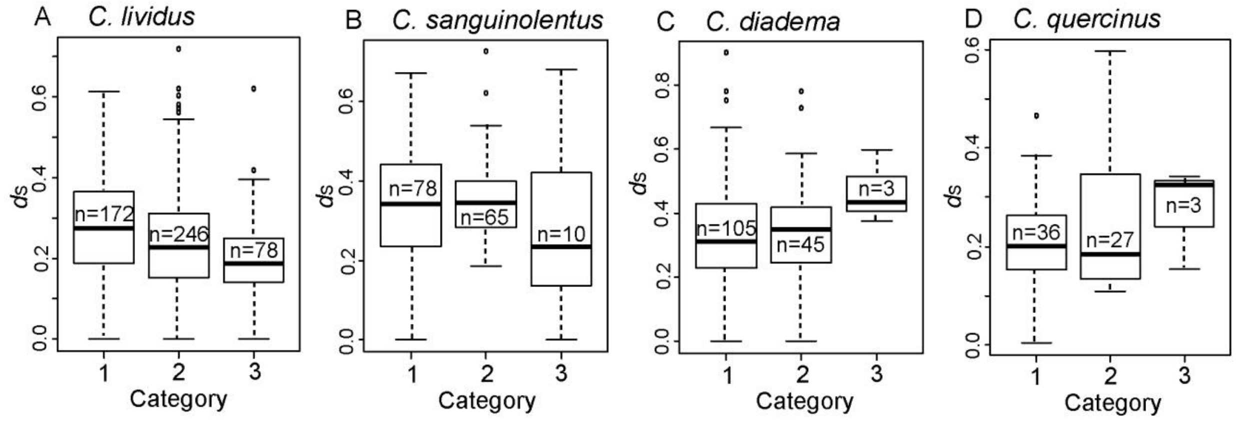

Supplement: Additional file 3 — Supporting table S1 and figures S1–S2. [file 1471-2148-14-123-S3.pdf]
